# Non-pharmacological interventions for schizophrenia—analysis of treatment guidelines and implementation in 12 Southeast European countries

**DOI:** 10.1038/s41537-022-00226-y

**Published:** 2022-03-01

**Authors:** Lidija Injac Stevović, Selman Repišti, Tamara Radojičić, Norman Sartorius, Sonila Tomori, Alma Džubur Kulenović, Ana Popova, Martina Rojnić Kuzman, Ilias I. Vlachos, Shukrije Statovci, Alexei Bandati, Antoni Novotni, Stojan Bajraktarov, Anca-Livia Panfil, Nadja Maric, Mirjana Delić, Nikolina Jovanović

**Affiliations:** 1 Faculty of Medicine, University of Montenegro; Psychiatric Clinic, Clinical Center of Montenegro, Podgorica, Montenegro; 2 Psychiatric Clinic, Clinical Center of Montenegro, Podgorica, Montenegro; 3 Association for the Improvement of Mental Health Programmes (AMH), Geneva, Switzerland; 4 University Hospital Center “Mother Teresa”, Tirana, Albania; 5 Clinical Center of University of Sarajevo, Sarajevo, Bosnia and Herzegovina; 6 Mental Health Center “Prof. Nikola Shipkovenski”, Sofia, Bulgaria; 7 Zagreb School of Medicine, Zagreb University Hospital Center, Zagreb, Croatia; 8 First Psychiatry Department, Eginition Hospital, National and Kapodistrian University of Athens, Athens, Greece; 9 Psychiatry Clinic, Prishtina, Kosovo; 10 Republican Dispensary of Addictions, Chișinău, Moldova; 11 University Clinic of Psychiatry, Skopje, North Macedonia; 12 County Emergency Clinical Hospital “Pius Brinzeu” Timisoara, Liaison Psychiatry Department, Bucharest, Romania; 13 Faculty of Medicine, UB Institute of Mental Health and Clinic for Psychiatry CCS, Belgrade, Serbia; 14 University Psychiatric Clinic, Ljubljana, Slovenia; 15 Queen Mary University of London, London, UK

**Keywords:** Schizophrenia, Human behaviour

## Abstract

This study aimed to analyze treatment guidelines of 12 SEE countries to identify non-pharmacological interventions recommended for schizophrenia, explore the evidence base supporting recommendations, and assess the implementation of recommended interventions. Desk and content analysis were employed to analyze the guidelines. Experts were surveyed across the 12 countries to assess availability of non-pharmacological treatments in leading mental health institutions, staff training, and inclusion in the official service price list. Most SEE countries have published treatment guidelines for schizophrenia focused on pharmacotherapy. Nine countries—Albania, Bosnia and Herzegovina, Bulgaria, Croatia, Greece, Moldova, Montenegro, North Macedonia, and Serbia—included non-pharmacological interventions. The remaining three countries—Kosovo (UN Resolution), Romania, and Slovenia—have not published such treatment guidelines, however they are on offer in leading institutions. The median number of recommended interventions was seven (range 5–11). Family therapy and psychoeducation were recommended in most treatment guidelines. The majority of recommended interventions have a negative or mixed randomized controlled trial evidence base. A small proportion of leading mental health institutions includes these interventions in their official service price list. The interventions recommended in the treatment guidelines seem to be rarely implemented within mental health services in the SEE countries.

## Introduction

The Southeast European (SEE) countries (Albania, Bosnia and Herzegovina, Bulgaria, Croatia, Greece, Kosovo (UN Resolution), Moldova, Montenegro, North Macedonia, Romania, Serbia, and Slovenia) share similar socioeconomic and political backgrounds. In the last three decades, these countries have gone through rapid socio-economic changes that have inevitably profoundly impacted both the populations’ mental health and development of mental health services^1,2^.

In these countries, attempts have been made to reform mental health care, improve patients’ human rights, and transition from asylum-based care to community-based care^3^. However, a recent evaluation of mental health care services in Central Europe and Eastern Europe suggests that mental health care across the region remains based around treatment in psychiatric hospitals with prescription medications^2^. This approach is particularly problematic for individuals diagnosed with schizophrenia because it leads to further social exclusion and inequality of this vulnerable group.

Schizophrenia is a severe and chronic illness with complex symptomatology, which affects up to 1% of the general population^4^. The symptoms can vary but often include hallucinations, delusions, disordered thinking, social withdrawal, alogia, and abulia^5^. Current treatment guidelines for schizophrenia published by the National Institute for Health and Care Excellence in the UK and the Schizophrenia Patient Outcomes Research Team in the USA suggest a combined-therapy approach including pharmacological (e.g. antipsychotics) and non-pharmacological interventions (e.g., talking therapy and family support)^6,7^.

Non-pharmacological interventions aim to improve the individual potential of people with mental disorders in their day-to-day life activities, including social and work domains^8^. They might have an important role in reducing the risk of relapse in schizophrenia^9^. In particular, psychosocial interventions (PIs) can be divided into three categories: (1) those based on education and support, (2) those including life and social skills training, and (3) problem-focused or symptom-focused interventions^10^. Another important group of interventions are psychotherapies (PTs) which can be defined as interpersonal interventions delivered by a trained clinician, and individualized to the client’s problem or modified so they can be suitable for delivery to a couple, family, or another group of clients^11^. PTs include two broad categories: system and individual interventions. It is important to note that a sharp boundary between PIs and PTs cannot be drawn. Most commonly recommended evidence-based non-pharmacological interventions for schizophrenia include cognitive-behavioral therapy (CBT), cognitive remediation, psychoeducation, social and coping skills, family interventions, and assertive community treatment (ACT) or case management^12^. Studies including non-pharmacological interventions as part of the multi-modal approach report mixed findings regarding their effectiveness^13^ as well as inconsistent implementation^14^.

Although a combined-therapy approach has been recommended in other regions, the topic of non-pharmacological treatment for individuals with schizophrenia standalone or as part of holistic approach to schizophrenia has received little research interest in SEE. We focused on non-pharmacological treatment in this study in order to bridge the gap in knowledge about its availability and implementation in SEE.

### Aims of the study

This study aimed to analyze treatment guidelines of 12 Southeast European countries to identify which non-pharmacological interventions are recommended for treating schizophrenia and if recommended interventions are based on robust evidence. The study also aims to explore the implementation of recommended interventions in each country.

## Results

### Treatment guidelines

The majority of participating countries (*N* = 9, 75%) have published treatment guidelines for schizophrenia. These documents were available and published under the following terms that can be used interchangeably: national guidelines, treatment guidelines, clinical instructions, therapeutic guidelines, practice guidelines, national clinical protocol, and instructions for treatment of schizophrenia. They included recommendations for both pharmacological and non-pharmacological treatment for schizophrenia. In Bosnia and Herzegovina, two treatment guidelines were identified, both covering pharmacological and non-pharmacological treatments. In Slovenia treatment guidelines for schizophrenia included only pharmacological treatment^15^. Finally, Kosovo (UN Resolution) and Romania do not have treatment guidelines for schizophrenia. Hence, 9 documents were analyzed in total. Table 1 shows recommended non-pharmacological interventions and indicators of implementation in each country.

**Table 1:** SEE countries with published treatment guidelines for non-pharmacological treatment of schizophrenia—recommended interventions and implementation indicators (availability of intervention, training staff, and whether the intervention is included in the official service price list).

Country		Treatment guidelines	Recommended interventions	Availability of intervention	Trained staff	Intervention included in the official service price list
1)	Albania	Ministry of Health (2018): Diagnostic and therapeutic care protocol for schizophrenia	1) Art therapy	No	No	No
			2) Cognitive-behavioral therapy (CBT)	No	No	No
			3) Family interventions	No	No	No
			4) Psychodynamic therapy	No	No	No
			5) Psychoeducation	No	No	No
			6) Social skills training	No	No	No
			7) Supportive therapy	No	No	No
2)	Bosnia & Herzegovina	1. Ministry of Health of the Canton of Sarajevo; Institute for Scientific Research and Development of University Clinical Center Sarajevo (2006): National guide for treating schizophrenia. 2.”Dr Mustafa Šehović Public Medical Center” and Association for Mutual Support and Mental Distress (FENIX) (2012): Manual for psychosocial interventions for persons with schizophrenia	1) Family interventions	Yes	Yes	Yes
			2) Professional rehabilitation	No	No	No
			3) Psychoeducation	Yes	Yes	No
			4) Social skills training	No	No	No
			5) Supportive psychotherapy	Yes	Yes	Yes
3)	Bulgaria	American Psychiatric Association / Bulgarian Psychiatric Association (1998): Practice guideline for the treatment of patients with schizophrenia	1) Assertive community treatment (ACT)	Yes	Yes	No
			2) Case management	Yes	Yes	No
			3) Cognitive-behavioral therapy (CBT)	Yes	Yes	No
			4) Cognitive remediation	No	No	No
			5) Family interventions	No	No	No
			6) Psychoeducation	Yes	Yes	No
			7) Self-help groups and organizations	Yes	Yes	No
			8) Social skills training	Yes	Yes	No
			9) Supported employment	Yes	Yes	No
4)	Croatia	Croatian Medical Association, Croatian Psychiatric Association, & Association for Mental Health Promotion (Svitanje; 2017): Psychiatric disorders encompassing psychosis and schizophrenia: Guidelines for psychosocial procedures and psychotherapy	1) Adherence therapy	No	No	No
			2) Art therapy	No	No	No
			3) Assertive community treatment	Yes	Yes	No
			4) Case management	Yes	Yes	No
			5) Cognitive-behavioral therapy (CBT)	No	No	Yes
			6) Cognitive remediation	No	Yes	No
			7) Family interventions	Yes	Yes	Yes
			8) Professional rehabilitation	No	No	No
			9) Psychoeducation	Yes	Yes	Yes
			10) Psychosocial interventions for maintaining optimal body weight	No	No	No
			11) Social skills training	Yes	Yes	Yes
5)	Greece	Greek Psychiatric Society (2015): Instructions for treating schizophrenia in Greece	1) Art therapy	Yes	N/A	Yes
			2) Cognitive-behavioral psychotherapy (CBT)	No	No	No
			3) Cognitive rehabilitation	No	No	No
			4) Compliance therapy	No	No	No
			5) Family interventions	Yes	Yes	Yes
			6) Psychoanalytic psychotherapy	No	No	No
			7) Psychoeducation	Yes	Yes	Yes
			8) Social skills training	Yes	N/A	Yes
6)	Moldova	Ministry of Health (2016): Schizophrenia first episode psychotic: National clinical protocol	1) Cognitive-behavioral therapy (CBT)	Yes	Yes	Yes
			2) Occupational therapy	No	No	No
			3) Psychosocial rehabilitation	No	No	No
			4) Family interventions	Yes	Yes	Yes
			5) Peer to peer support groups	No	No	No
			6) Psychoeducation	Yes	Yes	Yes
			7) Art therapy	No	Yes	Yes
			8) Psychomotor therapy	No	No	No
7)	Montenegro	Ministry of Health (2013): National guidelines of good clinical practice in treating schizophrenia	1) Cognitive-behavioral therapy (CBT)	No	No	No
			2) Family interventions	Yes	Yes	Yes
			3) Professional rehabilitation (programs for supported employment)	No	No	No
			4) Psychosocial interventions dealing with addiction problems as comorbid disorders	No	No	No
			5) Psychosocial interventions for maintaining optimal body weight	No	No	No
			6) Social skills training	No	No	No
8)	North Macedonia	Ministry of Health (2013): Instructions for the practicing of the evidence-based medicine within the treatment of schizophrenia	1) Cognitive-behavioral therapy (CBT)	No	No	Yes
			2) Family intervention	Yes	Yes	Yes
			3) Psychoanalytic psychotherapy	No	No	Yes
			4) Psychoeducation	Yes	Yes	Yes
			5) Social skills training	Yes	Yes	No
			6) Supported employment	Yes	Yes	No
9)	Serbia	Ministry of Health Working Group (2013): National guidelines of good clinical practice in treating schizophrenia	1) Art therapy	Yes	Yes	No
			2) Cognitive-behavioral therapy (CBT)	No	Yes	Yes
			3) Counseling and supportive psychotherapy	Yes	Yes	No
			4) Family psychotherapy	Yes	Yes	Yes
			5) Psychoeducation	Yes	Yes	No
			6) Self-help groups and associations	No	No	No
			7) Social skills training	Yes	N/A	No

*N/A* data not available.

Six out of 10 analyzed documents were published by the Ministry of Health of the respective country. The remaining four documents were published either by relevant professional organizations or by clinicians as follows. In Bosnia and Herzegovina, the *Manual for psychosocial interventions for persons with schizophrenia* (2012) was published by “Dr Mustafa Šehović Public Medical Center” and the Association for Mutual Support and Mental Distress (FENIX)^16^*. Practice guidelines for the treatment of patients with schizophrenia* was initially published by the American Psychiatric Association (APA) and was subsequently translated into Bulgarian and adopted by mental health policymakers in Bulgaria^17^. In Croatia, *Guidelines for psychosocial procedures and psychotherapy* (2017) was published by the Croatian Medical Association, Croatian Psychiatric Association, and Association for Mental Health Promotion (“Svitanje”)^18^. Finally, although not an official guideline, a widely accepted document containing recommendations and examples of good clinical practice in treating schizophrenia spectrum disorders in Romania is a monograph written by three Romanian clinicians with extensive experience in the treatment of psychotic disorders^19^.

The publishing year span was 20 years, starting with Bulgaria’s national guidelines published in 1998 and ending with the Albanian national guidelines published in 2018. On average, these documents were published 8.7 years ago (median value is 7 years).

As shown in Table 1, the number of recommended non-pharmacological interventions varied across countries and analyzed documents from 4 in North Macedonia to 11 in Bulgaria and Croatia. The average number of recommended interventions was 7. The 10 most recommended interventions in national guidelines were the following: CBT and family therapy/interventions (9 recommendations each), psychoeducation and social skills training (8 recommendations each), professional rehabilitation/supported employment (7 recommendations), and art therapy (6 recommendations). Supportive therapy and counseling, psychoanalytic/psychodynamic therapy, cognitive remediation/rehabilitation, occupational/ergotherapy were recommended in 3 out of 10 analyzed documents.

### Context of delivery and identified benefits of recommended interventions

Treatment guidelines were analyzed for additional information regarding the contextual and procedural recommendations of delivering non-pharmacological interventions for schizophrenia, along with empirical underpinning of their effectiveness.

*Albania—Diagnostic and therapeutic care protocol for schizophrenia* (Ministry of Health, 2018) contains very scarce information regarding non-pharmacological interventions for schizophrenia^20^. These therapeutic modalities are only listed as treatment programs that can be added to standard care offered to individuals with schizophrenia. The document does not contain definitions of interventions or their specific purpose.

*Bosnia and Herzegovina—National guide for treating schizophrenia* (Ministry of Health of the Canton of Sarajevo; Institute for Scientific Research and Development of University Clinical Center Sarajevo, 2006) does not discuss definitions and premises of specific interventions, but rather focuses on their aims and benefits^21^. *Manual for psychosocial interventions for persons with schizophrenia* contains a general introductory section about non-pharmacological interventions, their use, and usefulness in treating schizophrenia^16^. Furthermore, the document offers detailed descriptions of specific psychosocial interventions in the scope of psychoeducation.

*Croatia—Psychiatric disorders encompassing psychosis and schizophrenia: Guidelines for psychosocial procedures and psychotherapy* (Croatian Medical Association, Croatian Psychiatric Association, & Association for Mental Health Promotion, 2017) provides an overall description of various interventions as well as recommendations on when to use each of them^18^.

*Greece—Instructions for treating schizophrenia in Greece* (Ministry of Health, 2015) offers a detailed overview of various non-pharmacological interventions for schizophrenia. It contains information on when, where, and how it is best to deliver different interventions^22^.

*Moldova—Schizophrenia first episode psychotic: National clinical protocol* (Ministry of Health, 2016) contains several tables with recommendations on specific effects and how to deliver different non-pharmacological interventions, with no indications whether the recommendations are evidence-based^23^.

*Montenegro—National guidelines of good clinical practice in treating schizophrenia* (Ministry of Health, 2013) provides short descriptions of non-pharmacological interventions and the strength of evidence for their effectiveness based on the results of studies conducted in international settings^24^.

*North Macedonia—Instructions for practicing evidence-based medicine within the treatment of schizophrenia* (Ministry of Health, 2013) includes a list with several non-pharmacological interventions. Information provided is very scarce. This guideline refers to the degree to which each recommended intervention is supported by research evidence^25^.

*Serbia—National guidelines of good clinical practice in treating schizophrenia* (Ministry of Health Working Group 2013) were gathered to create and implement good clinical practice guidelines. The benefits of the recommended interventions were listed and supported by scientific references. Furthermore, the context of their delivery was explained^26^.

Table 2 shows *Kosovo* (UN Resolution) and *Romania*, two countries without published treatment guidelines. *Slovenia* has published treatment guidelines for schizophrenia however they do not recommend non-pharmacological interventions^15^. However, a handbook *Where and how to get help for mental illness?* provides a list of the recommended interventions coupled with their positive effects on mental health^27^. In *Romania*, a handbook of *Schizophrenia spectrum disorders* (2012) was written by Ienciu, Romosan, and Lazarescu^19^. The handbook cites various scientific sources when highlighting the importance of psychosocial rehabilitation techniques and methods in schizophrenia. It offers an overview of several interventions with detailed descriptions of their principles and usefulness.

**Table 2:** SEE countries without published treatment guidelines for non-pharmacological treatment of schizophrenia—available interventions and implementation indicators (trained staff sand whether the intervention is included in the official service price list).

	Country	Information on treatment guidelines	Interventions available in the leading institution	Trained staff	Intervention included in the official service price list
1)	Kosovo^a^	The document does not exist.	1) Family therapy	Yes	No
			2) Psychoeducation	Yes	No
			3) Social skills training	Yes	No
			4) Supportive therapy	Yes	No
2)	Romania	The document does not exist.	1) Art therapy	Yes	No
			2) Case management	Yes	No
			3) Cognitive-behavioral therapy (CBT)	Yes	No
			4) Ergotherapy	Yes	No
			5) Family therapy	Yes	No
			6) Psychoeducation	Yes	No
			7) Supportive therapy	Yes	No
3)	Slovenia	*Republiški strokovni kolegij za psihiatrijo* (2000): Priporocila in Smernice za zdravljenje z Shizofrenija^b^	1) Adherence therapy	Yes	Yes
			2) Art therapy	Yes	Yes
			3) Assertive community treatment	Yes	Yes
			4) Cognitive-behavioral therapy (CBT)	Yes	Yes
			5) Cognitive rehabilitation	Yes	Yes
			6) Ergotherapy	Yes	Yes
			7) Family therapy	Yes	Yes
			8) Occupational therapy	Yes	Yes
			9) Professional rehabilitation	Yes	Yes
			10) Psychodynamic therapy	Yes	Yes
			11) Psychoeducation	Yes	Yes
			12) Psychomotor therapy	Yes	Yes
			13) Psychosocial interventions dealing with addiction problems as comorbid disorders	Yes	Yes
			14) Psychosocial interventions focused on social inclusion	Yes	Yes
			15) Psychosocial interventions for maintaining optimal body weight	Yes	Yes
			16) Social skills training	Yes	Yes
			17) Supportive therapy	Yes	Yes

^a^UN Resolution.

^b^Slovenian treatment guidelines focus on medications only and non-pharmacological interventions are not mentioned.

#### Evidence base for recommended non-pharmacological interventions

Table 3 includes short descriptions of each recommended intervention from the analyzed treatment guidelines, country of origin, availability of treatment manual, duration of staff training, RCT evidence base, and evidence derived from SEE countries.

**Table 3:** Key characteristics and evidence base (RCTs and SEE effectiveness studies) for recommended interventions in the analyzed treatment guidelines.

	Recommended interventions	Short description	Country of origin (year)	Manual/guide for therapists	Duration of staff training	Randomized-controlled trial (RCT) evidence base	Effectiveness and/or implementation studies in SEE healthcare systems
1)	Art therapy	Form of psychotherapy that uses art media as its primary mode of communication.	United Kingdom (1960s)	N/A	2–3 years	Negative (RCT indicated that group art therapy was not more effective than activity groups or standard care; (Crawford et al.^35^)	N/A
2)	Adherence therapy^a^	Form of counselling that is mostly focused on advising the patient on how to take his/her medication properly.	United Kingdom (1998)	Gray et al.^36^	3–4 days	Mixed (Meta-analysis of six RCTs indicated that Adherence therapy was more effective in reducing psychiatric symptoms than usual treatment) (Gray et al.^37^)	N/A
3)	Assertive community treatment (ACT)	A person-centered form of community-based mental health care that includes support services for people with serious mental health illness.	USA (1970s)	Blokdyk^38^	2 days	Mixed (Meta-analysis of 6 RCTs and 11 observational studies showed that ACT yielded medium to large effects on symptoms, functioning, and well-being) (Norden, Malm, & Norlander^39^)	N/A
4)	Case management	Adjusting the course, form, and contents of mental health treatment to the patient’s needs.	USA (1980s)	Powell and Tahan^40^	N/A	Mixed (Meta-analysis of 40 RCTs showed that intensive case management may be valuable to individuals with high level of hospitalization in reducing hospitalization and increasing retention in care); Dietrich et al.^41^)	N/A
5)	Cognitive-behavioral therapy (CBT)	Form of psychological treatment which involves efforts to change thinking and behavioral patterns.	USA (1960s)	Smith et al.^42^	1–3 years	Mixed (Meta-analysis of 60 RCTs indicated that CBT may improve schizophrenia symptoms) (Jones et al.^43^)	N/A
6)	Cognitive remediation	This approach aims at improving neurocognitive functioning which could have a positive impact to psychosocial (everyday life) functioning.	Germany (1910s)	Haskins et al.^44^	3 months	Mixed (Meta-analysis of 26 RCTs indicated that Cognitive remediation produces moderate cognitive improvements); (McGurk et al.^45^).	Cognitive remediation linked with less negative symptoms and better quality of life, no effect on positive symptoms and social functioning (Trial data from Greece) (Rakitzi, et al.^28^)
7)	Compliance therapy	Form of counselling that is similar to the adherence therapy. It is more focused on making sure that the patient comes to appointments with the medical professional, as well as on lifestyle and dietary changes.	Australia & United Kingdom (1996)	Kemp & David^46^	N/A	Negative (single RCT;indicated that Complience therapy was not effective when compared to non-specific counselling, O’Donnell et al.^47^)	N/A
8)	Family interventions	A set of interventions that help family members to improve their mutual communication and resolve their conflicts in more adequate ways than before.	UK and USA (mid-20^th^ century)	Healios Ltd^48^	2 years	Mixed (meta-analysis of 25 intervention studies indicated that Family interventions can reduce the relapse rate by 20%) (Pitschel-Walz, et al.^32^).	N/A
9)	Occupational therapy (Ergotherapy)	Patients are involved in various activities, preferably designed by taking into account their abilities, interests, and needs.	USA (1910s)	American Occupational Therapy Association^49^	3 years (at least)	Negative (Pilot RCT indicating Occupational therapy appears to reduce positive and negative symptomatology) (Foruzandeh & Parvin^50^)	N/A
10)	Peer to peer support groups (including self-help groups and organizations)	Users of mental health services gather in order to support each other, because all of them went through some sort of mental health treatment as well as have experienced mental health issues.	France (late 18^th^ century)	N/A	N/A	Negative (No effects were found on reducing psychiatric symptoms, based on meta-analysis that included 18 trials) (Lloyd-Evans et al.^51^)	N/A
11)	Professional (vocational) rehabilitation	Its main aim is to train people for a suitable job, taking into account their disability. Other aims include helping them to maintain their jobs, as well as to develop professionally.	USA (early 1900s)	N/A	N/A	N/A	N/A
12)	Psychodynamic/ psychoanalytic therapy	Various forms of depth psychotherapy originated from the theory and practice of S. Freud.	Austria (1890s)	McWilliams^52^	2–4 years	Negative (no trials for psychoanalytic therapy and scarce data for psychodynamic approach) (Malmberg & Fenton^53^)	N/A
13)	Psychoeducation	Form of counselling where the patient is provided with information on the symptoms of his/her disease along with treatment options.	USA (1980)	Available manuals do not specifically refer to psychoeducation	N/A	Mixed (Systematic review of 44 trials indicated psychoeducation appears to reduce relapse, readmission and encourage medication compliance, as well as reduce the length of hospital stay); (Xia, Merinder & Belgamwar^33^)	Psychoeducation of patients’ family members was associated with greater compliance and reduction in hospitalization (Trial data from Greece) (Palli et al.^54^)
14)	Psychosocial interventions for maintaining optimal body weight	A set of interventions aimed at encouraging patients to change positive health behavior and lifestyle in a positive direction.	United Kingdom (2008)	Mooney at al,^55^	N/A	N/A	N/A
15)	Psychosocial interventions dealing with addiction problems as comorbid disorders	This kind of intervention is designed to address and treat substance-related issues that could also be detected in people with severe mental illness. It includes^56^: behavioral therapy approaches, family interventions, community reinforcement therapy, motivational enhancement therapy (MET), and CBT.	USA (2003)	N/A	N/A	N/A	N/A
16)	Psychosocial interventions focused on social inclusion (e.g. social recovery therapy)	The approach is focused on deinstitutionalization, stigma-reduction, and social (re)integration. Social inclusion is a broad term and can, for example, include supported employment.	EU (1990s)	Fowler et al.^57^	N/A	Positive (RCT indicated social recovery therapy plus early intervention services was associated with an increase in structured activity compared with early intervention services alone) (Fowler et al.^58^)	Social rehabilitation improved social functioning, self-esteem, and quality of life: case-controlled study from Croatia (Štrkalj-Ivezić et al.^29^)
17)	Psychomotor (body) therapy^b^	This is a holistic approach focused on the body expression which included not only physical, but also cognitive and emotional aspects.	Belgium, Germany, and Netherlands (1960)	N/A	500 h	N/A	N/A
18)	Social skills training	This kind of training includes the development of skills that are relevant for communication and interaction with the social environment (e.g., assertiveness, self-regulation, and empathy).	USA (1970)	Bellack et al.^59^	2 days	Mixed (Meta-analysis of 27 RCTs indicated that social skills training was associated with improvements in negative symptoms and general psychopathology (Turner et al.^60^)	N/A
19)	Supportive therapy	This technique overlaps with psychological/psychiatric counselling.	Europe and USA (mid-20^th^ century)	Novalis, Singer, & Peele^61^	It depends on the duration of training in other psychotherapies (e.g., CBT, psychoanalytic therapy, etc.)	Negative (Review of 24 trials indicated no difference compared to standard care) (Buckley et al.^62^)	N/A
20)	Supported employment		A form of employment for people with various disabilities, where an employment specialist, coworkers or other people help the person during his/her work engagement.	USA (1970s)	A manual specifically covering supported employment for people with schizophrenia does not exist.	N/A	Mixed (Review of 14 trials indicated supported employment was effective in improving several vocational outcomes, e.g. increasing length of competitive employment) (Kinoshita et al.^30^)

*N/A* not available (data or relevant study).

^a^The terms “adherence therapy” and “compliance therapy” are sometimes used interchangeably, referring to the same or similar psychosocial approach (as was the case in the expert survey). However, it seems their levels of effectiveness are not the same which is the reason they are presented separately in the table.

^b^Could be considered as a form of occupational therapy.

In total, 19 non-pharmacological interventions for individuals with schizophrenia were assessed. The majority of interventions were originally developed in the USA (*n* = 11) and manualized (*n* = 13). Seven interventions have a negative RCT evidence base meaning that, when compared to standard treatment/active control, the interventions were not effective. These interventions were: art therapy, compliance therapy (although adherence therapy, which was listed in the expert survey within the same item [*adherence/compliance therapy*] appeared to be effective), psychodynamic/ psychoanalytical therapy, psychosocial interventions for maintaining optimal body weight, psychosocial interventions dealing with addiction problems as comorbid disorders, psychosocial interventions focused on social inclusion, and supportive therapy. Two interventions—professional (vocational) rehabilitation and psychomotor (body) therapy—have never been studied in RCTs with individuals with schizophrenia.

The remaining 10 interventions have a mixed RCT evidence base. Family interventions, psychoeducation, social skills training, and cognitive behavioral therapy have stronger evidence-base compared to other interventions.

Only two effectiveness and/or implementation studies were conducted in SEE countries. First, an RCT exploring the effectiveness of cognitive remediation group therapy in patients with schizophrenia was conducted in Greece^28^. The findings indicated improvements in working memory and social perception during therapy and at 3-month follow-up. Second, a case-controlled study of 50 patients enrolled in a social rehabilitation program for 6 months compared with 50 patients on the waitlist reported improved social functioning, self-esteem, and quality of life^29^.

### Implementation of recommended interventions

As shown in Table 1, implementation indicators varied substantially across participating institutions. Most institutions did not offer all recommended interventions and even fewer interventions were included in the official service price lists. Some interventions that were on the price lists were not offered in services, possibly due to lack of trained staff to deliver these interventions. Most institutions included in the study faced a lack of clinical staff trained to deliver recommended interventions (Table 1). In Albania, none out of seven recommended interventions were available to patients in this country’s leading psychiatric institution. In other countries, the percentage of recommended interventions that were available and staff trained to deliver these interventions ranged from 14% in Montenegro to 78% in Bulgaria. In Albania and Bulgaria, recommended non-pharmacological interventions were not included in the official service price lists. The percentage of recommended interventions that were included in the official price list ranged from 14% in Montenegro to 67% in North Macedonia.

Treatment guidelines for non-pharmacological interventions for schizophrenia do not exist in Kosovo (UN Resolution), Romania, and Slovenia. Expert survey data showed that some were implemented in these countries (Table 2). In Kosovo (UN Resolution), the four following interventions were available to patients, although not included in their official service price list: family therapy, psychoeducation, social skills training, and supportive therapy. Seven different interventions were implemented in Romania: art therapy, case management, CBT, ergotherapy, family therapy, psychoeducation, supportive therapy. However, none of them are on the price list of the leading institution. Finally, in Slovenia, 17 different interventions were delivered to individuals with schizophrenia. All of them have been included in the price list of the respective institution.

## Discussion

This is the only study to date that explored treatment guidelines for individuals with schizophrenia in 12 SEE countries. The study looked specifically into non-pharmacological treatments for schizophrenia, which remains under researched topic globally.

The study showed that across the 12 SEE countries, 10 have published treatment guidelines for schizophrenia and 9 countries have guidelines for non-pharmacological interventions. Despite not having treatment guidelines in three countries, a range of non-pharmacological interventions were potentially available to patients with schizophrenia. Two interventions (family therapy and psychoeducation) were recommended by most treatment guidelines. The majority of recommended interventions had a mixed or negative RCT evidence base which is almost exclusively comprised of studies conducted outside SEE countries. The recommended interventions were insufficiently implemented in services and only a small proportion of leading institutions included them in their official service price list.

Treatment guidelines are essential for standardizing treatment and making it more consistent and efficient for people with specific conditions^30,31^. Guidelines should ideally be available online and local clinicians should be able to locate them easily. In our study, the majority of participating SEE countries had published treatment guidelines for schizophrenia, which seems to be an important step in improving the quality of provided treatment. Most guidelines included descriptions of non-pharmacological interventions, aims, benefits, and instructions for delivery which could be useful for busy clinicians aiming to integrate an evolving evidence-base into practice. However, most guidelines were more than five years old and therefore included recommendations that may be outdated. For example, at the publication of existing guidelines in 2015, the evidence for art therapy was considered to be strong, but new studies have since emerged contradicting this evidence. In discussion with national experts involved in the study, it seems that local clinicians often use the NICE guidelines available from the United Kingdom and follow international recommendations and consensus papers (e.g., published by European Psychiatric Association)^6^. While this approach may work for many clinicians, those clinicians who are not familiar with English language or know where to locate these documents will struggle to keep up to date with recommendations.

Two interventions, namely family therapy and psychoeducation (both developed in the USA), are recommended by most treatment guidelines from SEE countries. In 9 out of 12 countries, family therapy is available in the leading psychiatric institution, meaning that staff are trained and the intervention is on the service price list, indicating good implementation. The same is true for psychoeducation in 5 out of 12 countries. The evidence-base for these interventions seems to be stronger than for other interventions included in the studied guidelines. A meta-analysis of 25 trials examining family interventions—involving relatives in treatment and helping them to cope better with the patient’s illness—found a 20% reduction in relapse^32^. A systematic review of 44 trails found that psychoeducation appeared to reduce relapse and readmission, and encouraged medication compliance, as well as reduced the length of hospital stay^33^. Despite having a strong evidence base derived from studies in other regions, there is complete lack of research in SEE countries that systematically study the effectiveness and implementation of these interventions. This might be the main barrier for implementation of these interventions in mental health services in SEE countries. Additional challenges in implementation of treatment guidelines include lack of resources and/or inadequate use of current resources^34^.

The main strength of the study is that it provided a comprehensive overview of non-pharmacological treatment in the SEE region. The region remains the ‘blind spot on the global mental health map’^2^ and this study has the potential to fill the knowledge gap. The research team originates from SEE, which facilitated in-depth understanding of contextual factors related to developing and implementing treatment guidelines in participating countries. The study looked at published treatment guidelines and aspects of implementations as reported by experts in each participating country. Although this approach prevents us from deriving definitive conclusions about the implementation of recommended interventions in clinical practice, it can be used as a starting point for future interventional research as well as in-depth exploration of implementation barriers and facilitators. Some of the identified interventions could not be regarded as mutually exclusive and researchers struggled to allocate them into non-overlapping categories (e.g., Bulgarian national guidelines list individual therapy and CBT as two different non-pharmacological treatments for schizophrenia). The study focused on effectiveness data for identified interventions and evidence from qualitative studies was not explored. Despite these limitations, the study findings can contribute to the advancement of treatment of individuals with schizophrenia.

Treatment guidelines should be written by national experts and based on research data. The SEE countries need systematic studies on the effectiveness, cost-effectiveness, and implementation of non-pharmacological interventions for schizophrenia. As they provide important instruction in treatment of schizophrenia, treatment guidelines should be regularly updated in order to provide relevant data. Consistent re-evaluation of treatment guidelines is necessary and should be informed by newly acquired data coming from recent research.

Treatment guidelines should be updated with information relative to the most appropriate timing and type of psychosocial intervention based on illness stage (i.e., first episode vs chronic patients), clinical characteristics (i.e., positive symptoms vs predominantly negative symptoms), and general level of functioning. In addition, guidelines should be presented to clinicians with instructions on how to properly implement recommended interventions. We suggest continuous medical education complementary to treatment guidelines in order to provide up-to-date treatment direction.

Institutions should offer suitable trainings to their staff in order to facilitate implementation of evidence-base supported non-pharmacological treatments recommended in the treatment guidelines. Furthermore, we recommend training staff in additional evidence-based treatments that are not necessarily included in the treatment guidelines.

To conclude, most SEE countries have developed treatment guidelines for treating individuals with schizophrenia. The focus of these guidelines is mainly on pharmacotherapy, with less attention dedicated to discussion of the premises and benefits of non-pharmacological interventions. The majority of recommended non-pharmacological interventions have a mixed or negative RCT evidence base, which is almost exclusively comprised of studies conducted outside SEE countries. The recommended interventions seem to be poorly implemented in mental health services, which indicates large variation in delivery of mental health care across services. Existence of treatment guidelines is not sufficient for non-pharmacological treatment implementation. A substantial step towards implementation would be to include these non-pharmacological treatments into the official service price lists. Considering the limitations of the treatment guidelines and their implementation, it is clear that more research is warranted in the field. This study can be used as the first step in further research into developing evidence-based treatment guidelines in SEE countries, and ensuring their implementation in clinical practice.

## Methods

Treatment guidelines from each country were identified through online search or by country experts involved in the study. Desk and content analysis was used to ascertain fundamental features of identified treatment guidelines (title, publication year, and publisher) and to map recommended non-pharmacological interventions. Identified treatment guidelines were available in English or were translated into English by researchers (SR and TR).

Next, we assessed the evidence supporting recommended interventions and determined the source of evidence (e.g., SEE mental health services or elsewhere). The Cochrane Central Register of Controlled Trials and PubMed were searched for randomized controlled trials (RCTs) and the Cochrane Database of Systematic Reviews and PubMed were searched for meta-analyses and systematic reviews of identified non-pharmacological interventions published between 1 January 2010 and 31 December 2020. The latest or the most comprehensive systematic review/meta-analysis/RCT for each non-pharmacological intervention was selected. Selected publications were assessed for inclusion of patients from any of the SEE countries. A narrative overview of current evidence base for identified interventions was drafted. The randomized-controlled trial evidence base was defined as ‘negative’ (intervention compared to standard treatment/active control was not effective or single RCT with significant limitations or meta-analysis with no effect), ‘mixed’ (RCTs indicating the intervention is effective/non-effective) and ‘positive’ (at least one RCT without significant limitations showed the intervention was effective).

Several aspects of the implementation of recommended interventions in each country were explored using a survey of experts. The experts were psychiatrists working in the leading national psychiatric institution in the country involved in clinical and/or research activities related to schizophrenia. The leading institution was defined as the largest teaching/training institution in the participating country’s capital city. The rationale for choosing these institutions was that they are more likely to provide non-pharmacological interventions for people with schizophrenia than other institutions in or outside capitals. The experts were identified through established research and professional networks. Each expert has a longstanding career in working with patients with schizophrenia and is recognized as a such in their respective country. One expert per country was invited and the response rate was 100%. The survey consisted of 20 questions. For each intervention identified in the analyzed guidelines, experts were asked to provide information whether the intervention was available in their institution and if the recommended intervention is included in the official service price list. Whether an intervention is on the official service price list enables monitoring of intervention delivery, facilitates the process of staff training, and ensures that the interventions are delivered by the properly trained staff. The survey was designed by the core research group (LIS, NJ, NS, TR, SR) and piloted with experts from five countries (Bosnia and Herzegovina, Kosovo (UN Resolution), Montenegro, North Macedonia, and Serbia). Minor modifications concerning the formulation of questions and the survey’s layout were made before the survey was emailed to experts from the remaining seven SEE countries. Collected data were analyzed using descriptive statistics.

### Ethical approvals and informed consents

All procedures were approved by the ethics committees of the respective institutions in the countries participating in the IMPULSE project (within which the present study has been conducted): Bosnia and Herzegovina (Klinički Centar Univerziteta u Sarajevu— 03-02-4216, JU Psihijatrijska bolnica Kantona Sarajevo i JU Zavod za bolesti ovisnosti Kantona Sarajevo 02.8—408/19), Serbia (Medicinski fakultet u Beogradu—2650/XII-20 and Specijalna bolnica ‘Dr Slavoljub Bakalovic’ Vršac—01-36/1), Kosovo (Hospital and University Clinical Service of Kosovo—2019-85), Republic of North Macedonia (Medicinski Fakultet pri UKIM vo Skopje—03-24219), and Montenegro (JZU Klinicki Centar Crne Gore—03/01-29304/1, ZU Specijalna Bolnica za Psihijatriju “Dobrota” Kotor and JZU Dom Zdravlja “Dr Nika Labovic” Berane—01-47).

The participants have not signed any written informed consent form due to the nature of the present study, which was mostly based on mental health policy analysis. The participants (i.e. experts) were informants who provided us with necessary information on mental healthcare services in their institutions and countries.

### Reporting summary

Further information on research design is available in the Nature Research Reporting Summary linked to this article.

## Supplementary information

Reporting summary

## Data Availability

All the collected data are available upon reasonable request from interested parties.
